# Cell membrane components of *Brucella melitensis* play important roles in the resistance of low-level rifampicin

**DOI:** 10.1371/journal.pntd.0008888

**Published:** 2020-12-29

**Authors:** Xiaowen Yang, Tonglei Wu, Wenxiao Liu, Guozhong Tian, Hongyan Zhao, Dongri Piao, Hai Jiang, Qingmin Wu

**Affiliations:** 1 State Key Laboratory for Infectious Disease Prevention and Control, National Institute for Communicable Disease Control and Prevention, Chinese Center for Disease Control and Prevention, Beijing, China; 2 Key Laboratory of Animal Epidemiology and Zoonosis of the Ministry of Agriculture, College of Veterinary Medicine, China Agricultural University, Beijing, China; 3 College of Animal Science and Technology, Hebei Normal University of Science & Technology, Qinhuangdao, China; 4 Beijing Key Laboratory for Prevention and Control of Infectious Disease in Livestock and Poultry, Institute of Animal Husbandry and Veterinary Medicine, Beijing Academy of Agricultural and Forestry Sciences, Beijing, China; School of medicine, CHINA

## Abstract

*Brucella* spp. are facultative intracellular pathogens that can persistently colonize host cells and cause the zoonosis- brucellosis. The WHO recommended a treatment for brucellosis that involves a combination of doxycycline, rifampicin, or streptomycin. The aim of this study was to screen rifampicin-resistance related genes by transcriptomic analysis and gene recombination method at low rifampicin concentrations and to predict the major rifampicin- resistance pathways in *Brucella* spp. The results showed that the MIC value of rifampicin for *B*. *melitensis* bv.3 Ether was 0.5 μg / mL. Meanwhile, *B*. *melitensis* had an adaptive response to the resistance of low rifampicin in the early stages of growth, while the SNPs changed in the *rpoB* gene in the late stages of growth when incubated at 37°C with shaking. The transcriptome results of rifampicin induction showed that the functions of significant differentially expressed genes were focused on metabolic process, catalytic activity and membrane and membrane part. The *VirB* operon, β-resistance genes, ABC transporters, quorum-sensing genes, DNA repair- and replication -related genes were associated with rifampicin resistance when no variations of the in *rpoB* were detected. Among the *VirB* operons, VirB7-11 may play a central role in rifampicin resistance. This study provided new insights for screening rifampicin resistance-related genes and also provided basic data for the prevention and control of rifampicin-resistant *Brucella* isolates.

## Author summary

*Brucella* spp. are facultative intracellular pathogens that can persistently colonize host cells and cause the zoonosis- brucellosis. The WHO recommended a treatment for brucellosis that involves a combination of doxycycline, rifampicin, or streptomycin. Rifampicin-resistance related genes were screened by transcriptomic analysis and gene recombination method at low rifampicin concentrations and the major rifampicin- resistance pathways in *Brucella* spp were predicted. The results showed that the *VirB* operon, β-resistance genes, ABC transporters, quorum-sensing genes, DNA repair- and replication -related genes were associated with rifampicin resistance when no variations of the in *rpoB* were detected. Among the *VirB* operons, VirB7-11 may play a central role in rifampicin resistance.

## Introduction

*Brucella* spp. are facultative intracellular pathogens that can persistently colonize animal host cells and cause the zoonosis- brucellosis. The main symptoms of brucellosis are fever, sweating, weakness, and joint pain. Severe symptoms will make patients with brucellosis incapacitated. Brucellosis affects public health and safety and even economic development[1]. Based on biochemical characteristics and host preferences, twelve different species have been identified[2–6]. Most diagnosed human brucellosis cases to date have been caused by *B*. *melitensis*, *B*. *abortus*, *B*. *canis* and *B*. *suis*. If brucellosis patients are not diagnosed and treated in time, the infection might easily cause various complications, such as spondylitis, endocarditis, or encephalitis[7]. The number of human brucellosis cases exceeds 500,000 per year worldwide, and the incidence of human brucellosis in some endemic countries exceeds 100 per million [8]. Especially in the Middle East, the prevalence of brucellosis per million population is above 200, with the highest rate in Syria (1603.4). However, according to the World Health Organization (WHO), the actual incidence is more than 10–25 times that reported [9]. In China, the number of human brucellosis cases in 2018 was 39,296, which was slightly less than that in 2017 (40042 cases). In the past, the primary *Brucella* epidemic strains belonged to *B*. *melitensis* biovars 1 and 3 [10], while the main isolates in recent years belonged to *B*. *melitensis* biovar 3 [11].

*Brucella* spp. mainly exist in macrophages, and rifampicin and streptomycin are commonly used in clinical treatment [12]. In some cases, fluoroquinolones and macrolides can be used as alternatives [13]. The WHO recommended a treatment for brucellosis in 1989 that involves a combination of doxycycline- and rifampicin for six weeks or doxycycline alone for six weeks first, then in combination with streptomycin for 2–3 weeks [14]. This treatment is still recommended for brucellosis. Rifampicin belongs to the rifamycin group, which exerts its bactericidal effect by blocking bacterial RNA and protein synthesis. The known rifampicin resistance-related gene in *Brucella* spp. is *rpoB*, which encods a DNA-dependent RNA polymerase β subunit. Some results showed that variation in the *rpoB* gene caused reduced sensitivity to rifampicin [15]. Studies of *B*. *melitensis* and *B*. *abortus* have demonstrated that the highly variable region (520–580 sites) of the *rpoB* gene could affect the levels of rifampicin resistance [16]. Current studies have indicated that rifampicin-resistant *Brucella* spp. were found in countries [17–22] such as Turkey [23] and Egypt [24]. Meanwhile, rifampin-resistant isolates were also found in China in recent years [25].

The double dilution test method was used to determine the inhibition of the susceptibility of the bacteria to the drug, and the minimum inhibitory concentration (MIC) value was determined as the concentration of the drug in 1 mL that completely inhibited the growth of bacteria. In other words, the MIC value also indicated the sensitivity of these bacteria to the drug. As MIC breakpoints for rifampicin against *Brucella* had not yet been established, guidelines for slow-growing bacteria were used as an alternative. Based on the CLSI breakpoints for slow-growing bacteria (CLSI M100-S24), *Brucella* isolates were resistant to rifampin when the MIC value was ≥ 2 μg / mL. Except for the *rpoB* gene, no other rifampicin resistance genes have been reported. Thus, the aim of this study was to screen rifampicin resistance-related genes by transcriptomic analysis at low rifampicin concentrations and to predict the major rifampicin-resistance pathways in *Brucella* spp. The gene recombination method was also used to knock out some genes to confirm the role of these genes in rifampicin- resistance in *Brucella* spp. It is hoped that this study will provide new insights for screening rifampicin resistance-related genes and essential data for the prevention and control of rifampicin-resistant *Brucella* isolates.

## Results

### *B*. *melitensis* has an adaptive response and is resistant to low levels of rifampicin in the early stages of growth

**Fig 1:**
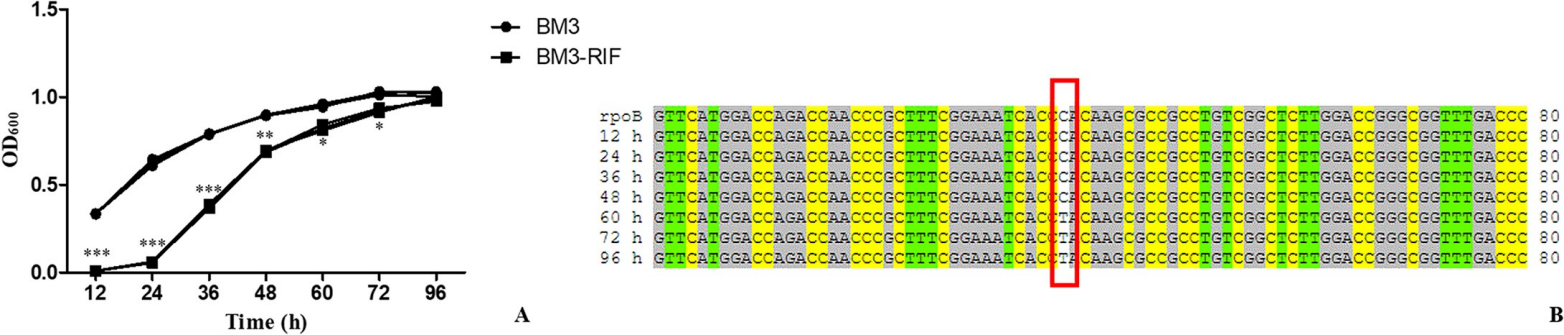
Growth characteristics and variations in the *rpoB* gene under normal and low-level rifampicin medium. (A) shows the growth characteristics of all samples. For the curve, BM3 was the parent strain grown without rifampicin and BM3-RIF was the parent strain grown with rifampicin. (B) shows the variation of parent strain grown with rifampicin in *rpoB* by sequencing at different time point.

The results MIC of rifampicin for *B*. *melitensis* bv.3 Ether was 0.5 μg/mL; based on this value and CLSI rifampicin breakpoints for slow-growing bacteria (MIC value ≥ 2 μg/mL), this study chose 1 μg/mL rifampicin for culture and transcriptome sequencing analysis. The growth characteristics of *Brucella* strains were determined in Brucella broth media without rifampicin and with 1 μg/mL rifampicin. Significant variations were observed between different media (Fig 1A). In the presence of rifampicin, the *Brucella* strains grew slowly for the first 24 h. At this time, the *Brucella* strains under normal medium were in the logarithmic phase, and those under rifampicin had barely replicated. At 24 h post-infection, the strains grown with rifampicin began to replicate, whereas the strains grown in normal medium were in the late logarithmic phase. After 96 h, the strains reached the plateau phase at low-level rifampicin. These results suggested that low-level rifampicin inhibited the in vitro growth of *Brucella* spp. at the early stages.

This study also detected the variation in the *rpoB* gene by sequencing at different times during growth. The results showed that no variations in the *rpoB* gene were observed during the first 48 h, while variation was observed in the *rpoB* gene at 60 h, and this variation was located at nucleic acid position 1606 (C→T) (Fig 1B).

Compared with the downloaded genome sequences, the *Brucella* strain in this study had 11 SNPs by whole genome sequencing. There were 8 SNPs before variations found in the presence of 1 μg/mL rifampicin, while 11 SNPs were found after variations were observed (Table 1). Seven SNPs were common among all samples. Compared with the downloaded genome sequences, the SNP at position 140902 of chromosome 1 was detected in the normal medium but not in the medium with rifampicin. This study also detected the SNP at position 832186 of chromosome 2 in the medium with rifampicin but not in the normal medium. Importantly, the SNP at position 187170 of chromosome 1 was only identified in the sample in the medium supplemented with rifampicin when the variation in the *rpoB* gene was observed. The above results showed that *B*. *melitensis* had an adaptive resistance response to low levels of rifampicin in the early stages of growth. At the same time, the SNPs in the *rpoB* gene changed in the late stages of growth when incubated at 37°C with shaking.

**Table 1:** SNPs in *B*. *melitensis* under low-level rifampicin and normal media.

Chromosome	BM3T	BM31 (no variations)	BM31 (with variations)	Annotation
Etherchromosome1	127451	127451	127451	intergenic region#
Etherchromosome1	140902	/	/	intergenic region
Etherchromosome1	172495	172495	172495	intergenic region
Etherchromosome1	/	/	187170*	missense variation
Etherchromosome1	815415	815415	815415	intergenic region
Etherchromosome1	998263	/	/	synonymous variation
Etherchromosome1	1089097	/	1089097	missense variation
Etherchromosome1	1380326	/	1380326	missense variation
Etherchromosome2	84544	84544	84544	intergenic region
Etherchromosome2	117849	117849	117849	intergenic region
Etherchromosome2	531185	531185	531185	intergenic region
Etherchromosome2	/	832186	832186	frameshift variation
Etherchromosome2	1079291	1079291	1079291	intergenic region

Note: All samples were compared with B. melitensis bv.3 Ether (reference genome). BM31 (no variations) is no variation had been observed in *rpoB* gene, and BM31 (no variations) is variations had been observed in *rpoB* gene.

* This SNP was the variation of *rpoB* gene located at nucleic acid position 1606.

# Intergenic region shows the SNP was located between genes; missense/ synonymous/ frameshift variation shows the SNP was located in the gene.

### Genome-wide identification of *B*. *melitensis* genes that respond to rifampicin conditions before the variation in *rpoB* was observed

**Fig 2:**
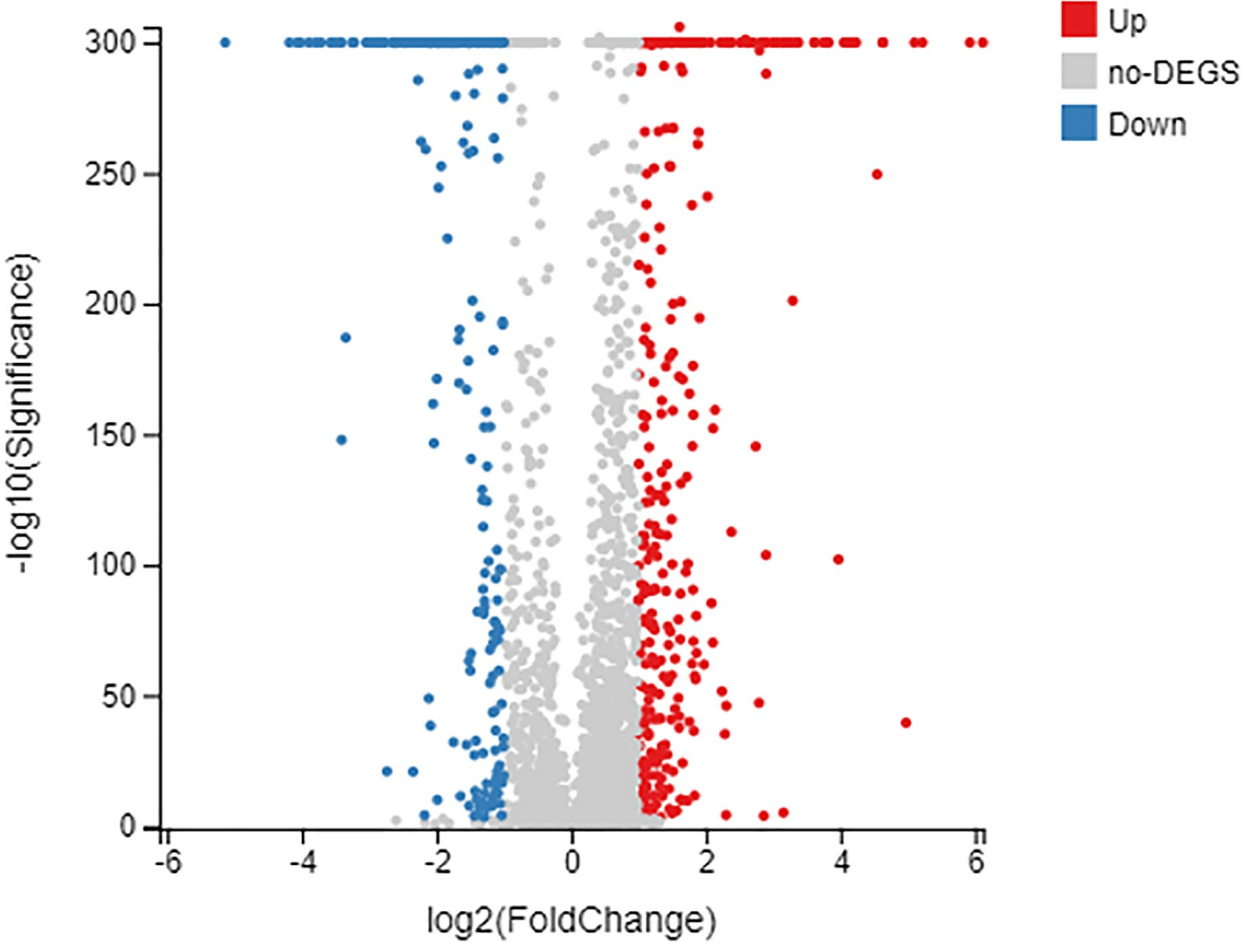
Differentially expressed genes identified by transcriptome analysis of low-level rifampicin.

This study analyzed the effects of rifampicin on gene expression in *B*. *melitensis* without variation in the *rpoB* gene using transcriptome sequencing. The total clean bases of the control (BM3T) and rifampicin-challenged (BM31) samples were 6.66 and 6.49 Gb, respectively. The RNA-seq results showed that exposure to rifampicin led to significantly changed transcript levels of genes with various functions. Overall, 122 genes were up- or downregulated (Fig 2) (S4 Table).

### Different gene expression categories and pathway analysis

**Fig 3:**
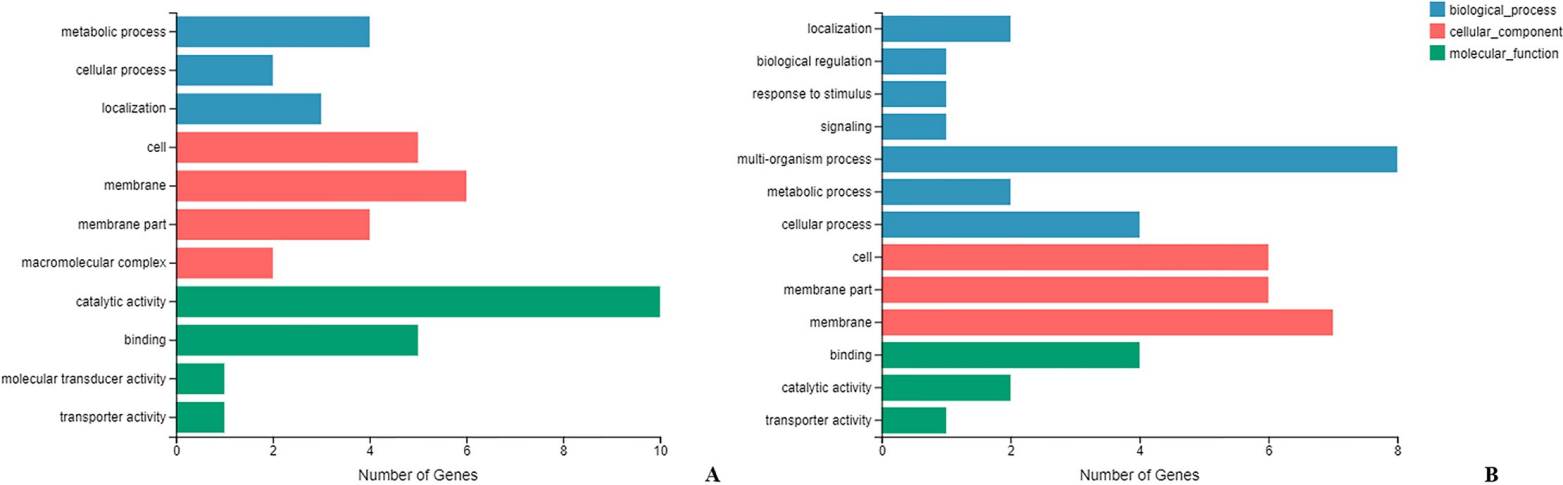
GO annotation of differentially expressed genes assigned to three GO categories. (A) shows the categories of upregulated genes, (B) shows the categories of downregulated genes. Blue color indicated the biological process function, including metabolic process, localization, biological regulation, response to stimulus, signaling, multi-organism process, cellular process; red color indicated the cellular component function, including macromolecular complex, cell, membrane part, membrane; green color indicated molecular function, including binding, catalytic activity, transporter activity, molecular transducer activity.

Biological process, molecular function, and cellular component were the three categories of gene ontology (GO) analysis. The gene expression analysis showed that the gene functions of the significantly upregulated gene (21 genes) were enriched in molecular function and cellular component, especially in metabolic process (category P), catalytic activity (category F) and membrane and membrane part (category C) (Fig 3A). The gene expression analysis showed that the gene functions of the significantly downregulated gene (20 genes) were also enriched in molecular function and cellular component, especially in multi-organism process (category P) and membrane and membrane part (category C) (Fig 3B).

**Fig 4:**
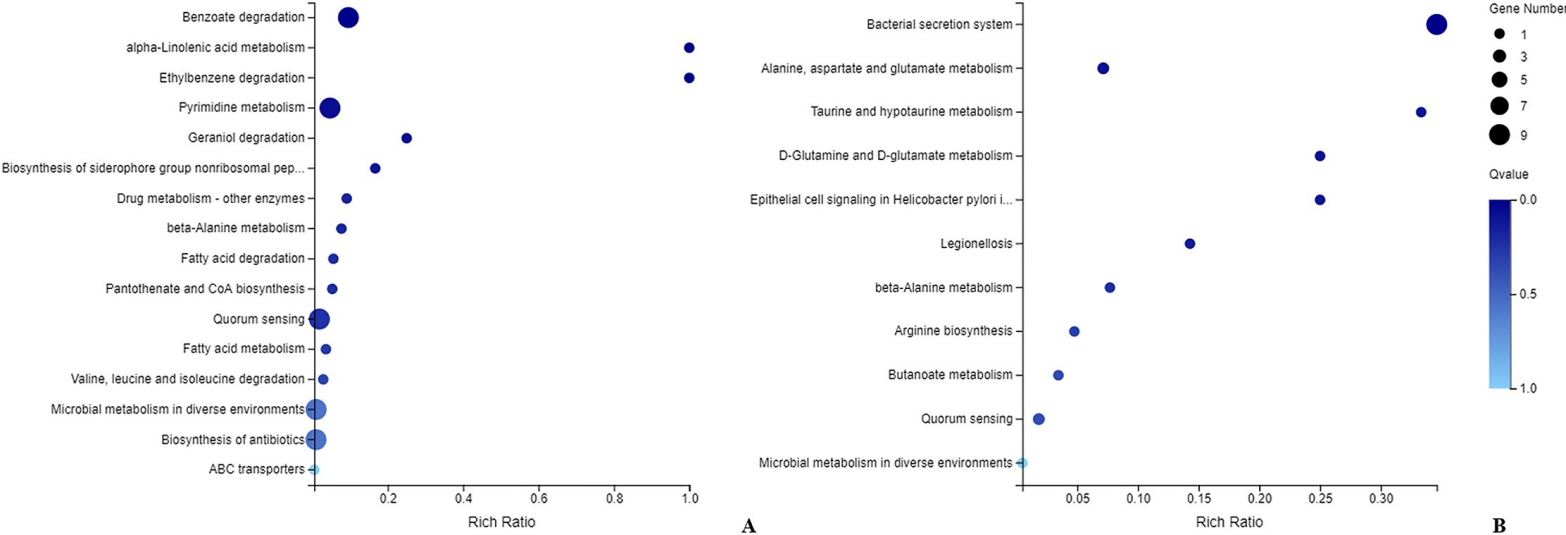
KEGG distribution pattern of differentially expressed genes. (A) shows the distribution of upregulated genes, and (B) shows the downregulated genes. The ordinate denotes the pathway name, the abscissa denotes the rich factor, the size of the dot indicates the number of differentially expressed genes in this pathway, and the color of the dot corresponds to different Q-value ranges.

KEGG pathway analysis demonstrated that the significantly upregulated genes were enriched in benzoate degradation, pyrimidine metabolism, quorum sensing, microbial metabolism in diverse environments and biosynthesis of antibiotics (Fig 4A), whereas the significantly downregulated genes were primarily involved in the bacterial secretion system (Fig 4B).

### Cell membrane components of *B*. *melitensis* play essential roles in resistance to low rifampicin concentrations before the observed variation in the *rpoB* gene

**Fig 5:**
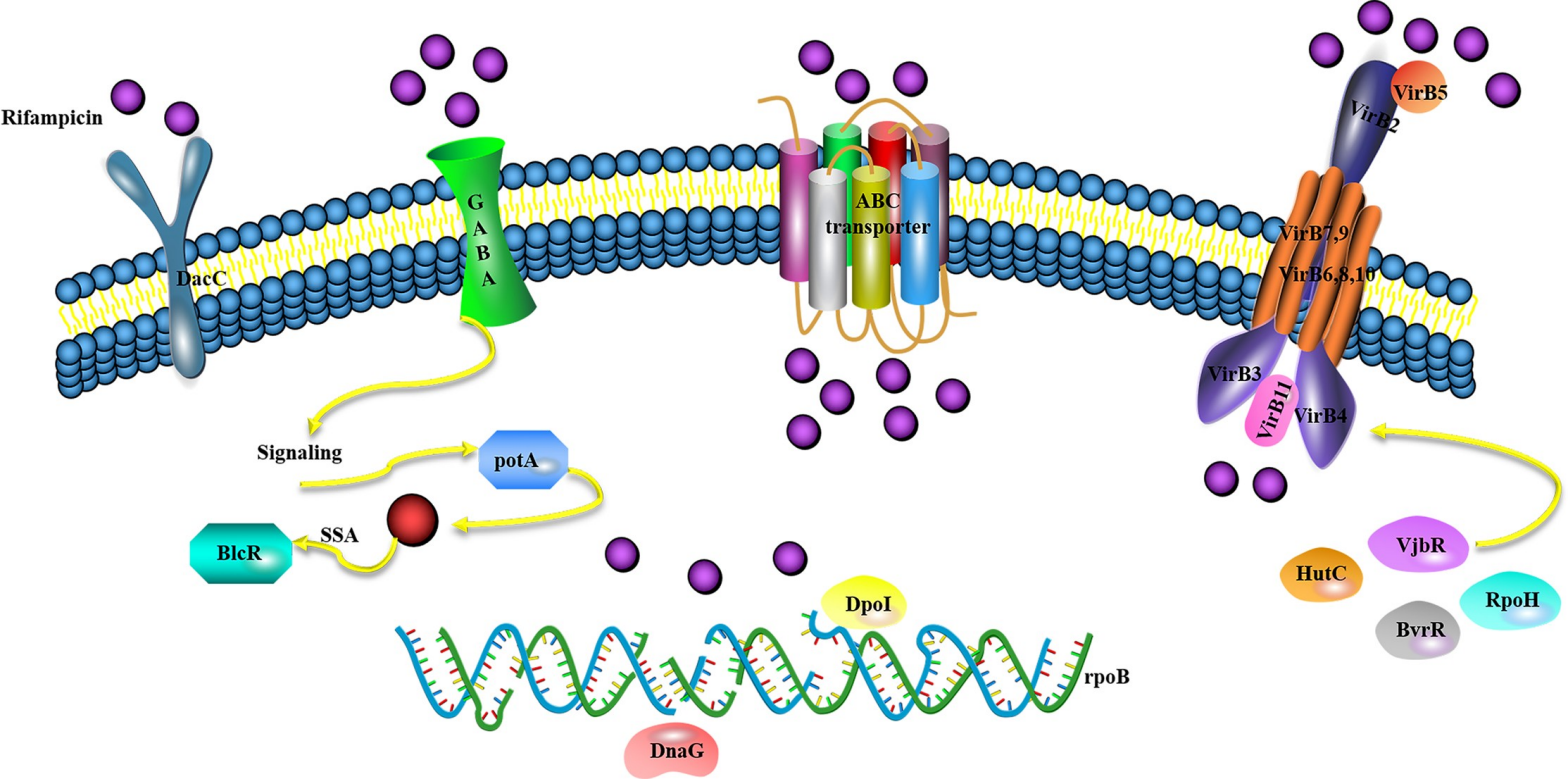
Hypothesis model of the rifampicin resistance mechanism in *B*. *melitensis*.

The most exciting results were that 12 genes associated with the type IV secretion system (T4SS) were enriched and downregulated by rifampicin without variations in *rpoB*, including the *VirB*1-12 gene. Among the functions of cell membrane components, the quorum-sensing protein *potA*, β-resistance *DacC*, and ABC transporter *AapJMQ* genes were upregulated. In the cytoplasm, the T4SS regulation factor *vjbR*, *HutC*, *BvrR* and *RpoH*-like heat shock sigma factor were downregulated; while the DNA repair protein *DpoI* and DNA replication protein *DnaG* were upregulated. Based on the transcriptome results in this study, the following low rifampicin resistance mechanism of *Brucella* spp. was suggested: 1) Rifampicin exposure stimulates the outer membrane of cells to produce GABA, leading to an increase in the expression of quorum-sensing protein *potA*, which activates the quorum-sensing system. Furthermore, *potA* may regulate the physiological characteristics of *Brucella* spp. 2) the ABC transporter *AapJMQ* genes were upregulated, and their role may be to excrete rifampicin. 3) The expression of the T4SS regulation factor *vjbR* [26], *HutC* [27], *BvrR* [28] and *RpoH*-like heat shock sigma factor [29] were down-regulated, while the expression of the *VirB* operon was also downregulated, which led to a reduction in the secretory system. These changes may lead to less rifampicin intake. 4) Other known resistance-related genes such as *DacC* were upregulated for rifampicin resistance. 5) The expression of the DNA repair protein *DpoI* and DNA replication protein *DnaG* in the cytoplasm was upregulated, and these proteins may be used for *rpoB* variation or self-repair (Fig 5).

### The role of the *Vir*B operon in low rifampicin resistance

**Fig 6:**
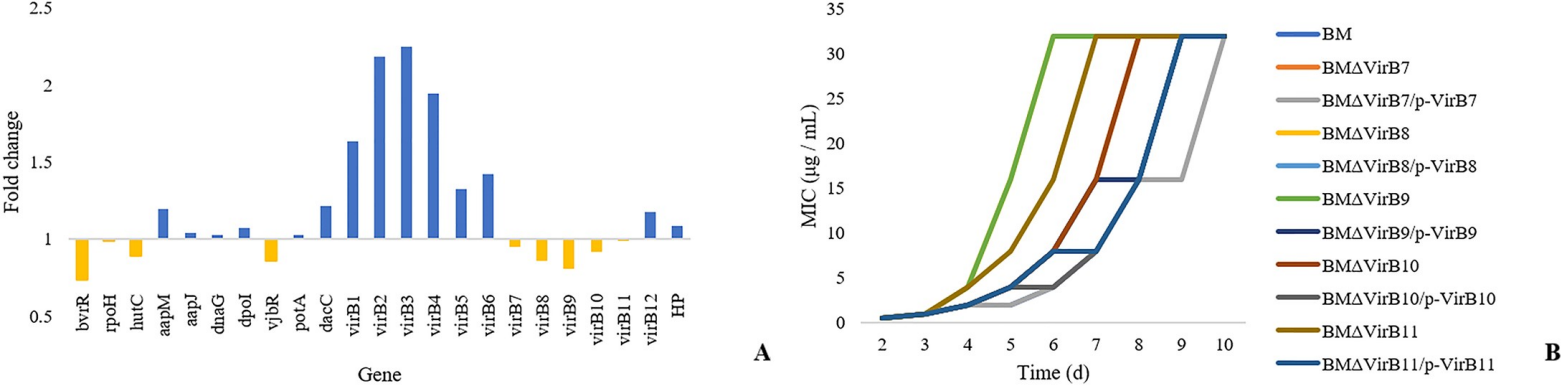
MIC values of rifampicin for the gene deletion and parent strains cultured for 10 d. (A) shows the expression levels of the *VirB* genes by RT-PCR, (B) shows the MIC values of the gene deletion and parent strains.

The expression levels of genes in the *VirB* operon were evaluated under low rifampicin conditions (Fig 6A). The results showed that the expression of *VirB*1-6 and *VirB*12 was induced by low rifampicin, while the expression of *VirB*7-11 was inhibited in the samples treated with low rifampicin compared with the controls.

*VirB*7-11 gene deletion strains named BMΔ*VirB*7- BMΔ*VirB*11 were constructed in this study. The growth characteristics of the gene deletion and parent strains were determined in Brucella broth medium in 96-well plates. No significant variations were observed. The gene deletion and parent strains were cultured under different rifampicin concentrations. Under rifampicin conditions, all MIC values of the strains were not different. After 3 d, the MIC values of the gene deletion strains were higher than those of the parent strain (Fig 6B). The There was no variation in *rpoB* during the culture by sequencing. We also constructed the *virB*1-6 and *virB* 12 genes deletion mutants and named BMΔ*VirB*1- BMΔ*VirB*6 and BMΔ*VirB*12. The growth characteristics of the gene deletion and parent strains were determined and no significant variations were observed. Under rifampicin conditions, all MIC values of the strains were not different compared with the parent strain.

The complemented gene deletion mutants named BMΔ*VirB*7/p-*VirB*7- BMΔ*VirB*11/p-*VirB*11. There was no significant difference in growth characteristics between the complemented gene deletion mutants and parent strains. Meanwhile, although the complemented gene deletion mutants showed variations in MIC values of rifampicin compared with parent strains, it also demonstrated lower MIC values compared with the gene deletion strains at the same time points. It was also found that all MIC values of the strains were not different after 10 days. These results showed that *VirB*7-11 played important roles in rifampicin adaptive resistance in *B*. *melitensis*.

## Discussion

As drug sensitivity testing of *Brucella* spp. needs to be performed in a BSL-3 laboratory, little work has been done on sensitivity testing for a long time. *Brucella* spp. are generally considered to be sensitive to common clinical drugs. Some results showed that *rpoB* was related to the rifampicin resistance of *Brucella* strains. This study found that *rpoB* was mutated at different times under sharking or incubator culture. The *rpoB* gene was mutated after 48 h by shaking culture, while no variation was detected until one week by incubator culture under low rifampicin. Meanwhile, multiple low-level rifampicin induction tests found that the *rpoB* gene was usually mutated at position 1606, but sometimes it is mutated at position 1576. The rifampicin resistance level of this variation was consistent with the variation at position 1606 in *rpoB*. Position 1576 is also located in the highly variable region of the *rpoB* gene. In this study, we also found that no variations in the *rpoB* gene were observed during the first 36 h. While at 48 h, 1–2 colonies were occasionally mutated among 10 colonies. The variation of the *rpoB* gene of 8–10 colonies were observed at 60 h.

The T4SS is a multi-protein complex and is present in many gram-negative bacteria, such as *rhizobium*, *Helicobacter pylori*, *Legionella pneumophila*, and *Brucella* spp. [30]. T4SS allows substrates to pass through the cell membrane and allows the release of secreted proteins into the outside of the cell. The T4SS is encoded by the *VirB* operon, which plays an important role in conjugation and DNA uptake and release and is also an important virulence factor for *Brucella* [31]. The *VirB* operon includes 12 genes, *VirB* 1–12 [32]. Different genes in the *VirB* operon have different functions, and *VirB*1 may affect other *VirB* genes. The *VirB*6, *VirB*7, and *VirB*10 proteins are transmission signals of bacterial transmembrane proteins. The *VirB*4 and *VirB*11 proteins are coupled with the *VirD*4 protein and allow the ATPase to enter from the surface to the membrane. The *VirB*2 and *VirB*5 genes may be pilus structure proteins. *VirB*8 plays a major role in the type IV secretion system, mainly forming a population on the cell membrane with *VirB*9 and *VirB*10 [33]. The functions of *VirB*3 and *VirB*12 are not precise, and they may be related to the assembly of the complex [34]. This study showed that *VirB*7-11 played essential roles in rifampicin adaptive resistance in *B*. *melitensis* after cultur for 3 d. The MIC values of BMΔ*VirB*7-11 were higher than those of the parent strain after culture for a long time. This study also constructed *VirB*1-6 and *VirB*12 gene deletion strains. All MIC values of the strains were no different during the experiment.

Compared with the CARD database[35], *DacC* was the known drug resistance gene among significantly differentially expressed genes. *DacC* encods penicillin-binding protein 6 (PBP6)[36]. PBP6 contains the β-lactam/penicillin-binding domain motif sequences[37], is required for cell components and provides some resistance to penicillin[38]. Following its debut in 1967, rifampicin has become a common clinical drug in the treatment of tuberculosis, leprosy and so on. Rifampicin specifically inhibits bacterial RNA polymerase. However, resistance to rifampicin was reported shortly after its introduction in the clinical treatment. Studies in *Escherichia coli* helped to define the molecular mechanism of rifampicin- resistance, demonstrating that resistance is mostly due to variations in *rpoB* encoding the RNA polymerase β chain[39]. The new study showed that some *Brucella* isolates were resistant to rifampicin and had variations in *rpoB*, suggesting that there were other rifampicin resistance genes in *Brucella* spp. Using transcriptome sequencing under low-level rifampicin conditions, this study found that the *VirB* operon, β-resistance genes, ABC transporter, quorum-sensing genes, DNA repair- and replication- related genes were associated with rifampicin resistance when no variations in *rpoB* were detected.

In conclusion, this study used transcriptome sequencing under low-level rifampicin conditions and gene recombination methods. The results showed that the MIC value of rifampicin for *B*. *melitensis* bv.3 Ether was 0.5 μg/mL. *B*. *melitensis* had an adaptive response to the resistance of low rifampicin in the early stages of growth, while the SNPs changed in the *rpoB* gene in the late stages of growth when incubated at 37°C with shaking. The transcriptome results of rifampicin induction showed that the functions of significant differentially expressed genes were focused on metabolic process, catalytic activity and membrane and membrane part. The *VirB* operon, β-resistance genes, ABC transporters, quorum-sensing genes, DNA repair- and replication- related genes were associated with rifampicin resistance when no variations in *rpoB* were detected. Among the *VirB* operons, *VirB*7-11 may play a central role in rifampicin resistance. This study provided new insights for screening rifampicin resistance-related genes and provided basic data for the prevention and control of rifampicin-resistant *Brucella* isolates.

## Materials and methods

### *Brucella* strain and genome in this study

The genome, amino acid, and nucleic acid sequences of *B*. *melitensis* bv.3 Ether were downloaded from the NCBI RefSeq database (ftp://ftp.ncbi.nlm.nih.gov/genomes/all/GCF/000/740/355/GCF_000740355.1_ASM74035v1). A cultured and inactivated sample of *B*. *melitensis* bv.3 Ether were obtained in a BSL-3 laboratory of National Institute for Communicable Disease Control and Prevention of the Chinese Center for Disease Control and Prevention.

### Rifampicin susceptibility testing

The susceptibility of a *Brucella* sp. strain to rifampicin susceptibility was tested by the double dilution method according to CLSI guidelines. Rifampin was double diluted from 0.125–256 μg / mL in 96-well plates. Strains were suspended in saline water to a 0.5 McF turbidity, and suspended in *Brucella* broth adjusted to pH 7.1 ± 0.1 (BD, USA). The plate was cultured at 35 ± 2°C with 5% CO_2_ for 48 h. On the same plate, the negative and positive controls were set. The quality control strain was Streptococcus pneumoniae ATCC 49619 (refer to CISL_M45 (2016)).

### In vitro growth characteristics in *Brucella* broth medium

For the growth analysis of the *Brucella* strain in presence of different rifampicin concentrations in vitro, one colony of the *Brucella* strain was inoculated into 3 mL of *Brucella* broth medium and cultured for 24–48 h at 37°C in an incubator shaking at 200 rpm. Subsequently, the cultures were adjusted to the same concentration (OD_600_ ≈ 1.0) and used for growth curve analysis. A 50 μL sample of the *Brucella* culture was inoculated into 5 mL of Brucella broth medium with different concentrations of rifampicin. The cultures were incubated at 37°C with shaking at 200 rpm, and the OD_600_ value was determined at different time points. These strains were tested in triplicate in two independent experiments.

### RNA extraction and transcriptome

Before extraction, two volumes of RNAprotect Bacteria Reagent (Qiagen, DEU) were added directly to one volume of culture sample. RNA was extracted from the *Brucella* strains under different concentrations of rifampicin using TRIzol (Invitrogen, USA) according to the manufacturer’s instructions and treated with DNase (TaKaRa Bio, JPN) before reverse transcription to remove DNA contamination. Total RNA was dissolved in diethylpyrocarbonate (DEPC)-treated water, and the concentration and purity of the total RNA were estimated by NanoDrop and Aligilent 2100. The samples were sent to Huada Gene Co., Ltd. (China) for sequencing and transcriptome analysis.

To obtain more accurate and reliable results in subsequent bioinformatics analysis, the raw data were treated in the following manner: 1) reads with a certain proportion of low quality(≤20) bases(40% as default) were removed; 2) reads with a certain proportion of Ns (40% as default) were removed;3) adapter contaminations were removed; 4) duplication contaminations were removed to obtain clean data. Bowtie2[40] was used to align the reads with the reference genome. RSEM[41] software was used to calculate gene expression levels. Then, cluster and functional analyses were performed based on the different gene expression levels.

### qRT-PCR

cDNAs were synthesized by extracting total RNA using the PrimeScript RT Reagent Kit (TaKaRa Bio, JPN.) according to the manufacturer’s instructions. The reverse transcription product was stored at -20°C. PCR was performed with the primers shown in S1 Table to evaluate gene expression. The value obtained by the relative quantitative method (2^-△△Ct^) was used to compare the expression level of the reference gene at different rifampicin concentrations, and the 16S rRNA expression level in *B*. *melitensis* was used as a reference to normalize all values. Three replicate wells for each gene were evaluated, and the results presented in this paper represent the averages from at least three separate experiments.

### Whole genome sequencing

Bacterial genomic DNA was extracted using the Wizard Genomic DNA Purification kit (Promega, USA) according to the manufacturer’s instructions. Library preparation was performed using the Nextera XT Library Prep kit (Illumina, USA) according to the manufacturer’s manual. The libraries were sequenced using an Illumina/Solexa sequencing analyzer to 100-fold (100×) genome coverage at the Beijing Genomics Institute (BGI) (Shenzhen, China).

### SNPs identification and annotation

SNPs and indels identification and annotation were performed as described in a previous study [42] with minor modifications. We removed the SNPs with a quality score of less than 1000. All SNPs were identified by PCR amplification and sequencing.

### PCR assay of the *rpoB* gene

The highly variable region and whole sequence of the *rpoB* gene were amplified using primers designed by Vector NTI (www.invitrogen.com/VectorNTI). Sequencing and data analysis were conducted as previously described[43] with some modifications. The liquid culture was streaked on a plate, which was incubated at 37°C for 5 days and then 10 colonies were examined at each time points.

### Construction of gene deletion mutants

Gene deletion mutants were constructed using a recombination system with pBluescript_II phagemid vectors. The primers are shown in S2 Table. First, approximately 1050 bp of the chloromycetin resistance gene was amplified from plasmid pKD3, including the flippase recognition target (FRT). Second, the 500 bp flanking either side of the gene were amplified from *B*. *melitensis* bv.3 E ther genomic DNA with primers, gel-purified, and used as templates for a second round of overlapping PCR. Then, the fragments were cloned into pBluescript_II to generate the recombinant plasmid. Finally, the recombinant plasmid was introduced into *B*. *melitensis* bv.3 Ether via electroporation. Colonies that were sensitive to ampicillin (Amp) but not sensitive to chloromycetin (CM) were selected. The gene deletion mutants were then verified by PCR and sequencing analysis.

### Construction of the complemented gene deletion mutants

The complete gene and its operon promoter sequences were amplified (S3 Table) in order to construct the complemented strain. The fragment was recombined into the pBBR1mcs-2 plasmid. After confirmed by sequencing, the recombinant plasmid was subsequently electroporated into the gene deletion mutant. The complemented gene deletion mutant strains were selected on the *Brucella* agar medium containing kanamycin. Finally, the selected complemented strains were confirmed through PCR amplification.

### Statistical analysis

Statistical analyses were performed using Excel and SPSS. A *P* value < 0.05 was considered significant when using a one-way analysis of variance (ANOVA). The heatmap and other figures were drawn by R software.

## Supporting information

S1 Table RT-PCR primers in this study. (XLSX)

S2 Table Primers used for construction of the gene mutant in this study. (XLSX)

S3 Table Primers used for construction of the complemented gene deletion mutants in this study. (XLSX)

S4 Table Transcriptome results of different expression genes. (XLSX)
